# A systematic approach for testing expression of human full-length proteins in cell-free expression systems

**DOI:** 10.1186/1472-6750-7-64

**Published:** 2007-10-03

**Authors:** Claudia Langlais, Birgit Guilleaume, Nadja Wermke, Tina Scheuermann, Lars Ebert, Joshua LaBaer, Bernhard Korn

**Affiliations:** 1 MRC Toxicology Unit, Protein Profiling Group, Hodgkin Building, Lancaster Road, Leicester, LE1 9HN, UK; 2 German Ressource Center, Im Neuenheimer Feld 515, D-69120 Heidelberg, Germany; 3 Institute of Proteomics, Harvard Medical School, 240 Longwood Avenue, Boston, MA 02129, USA; 4 German Cancer Research Center, Genomics & Proteomics Core Facilities, Im Neuenheimer Feld 515, D-69120 Heidelberg, Germany

## Abstract

**Background:**

The growing field of proteomics and systems biology is resulting in an ever increasing demand for purified recombinant proteins for structural and functional studies. Here, we show a systematic approach to successfully express a full-length protein of interest by using cell-free and cell-based expression systems.

**Results:**

In a pre-screen, we evaluated the expression of 960 human full-length open reading frames in *Escherichia coli *(*in vivo *and *in vitro*). After analysing the protein expression rate and solubility, we chose a subset of 87 plasmids yielding no protein product in *E. coli in vivo*. These targets were subjected to a more detailed analysis comparing a prokaryotic cell-free *E. coli *system with an eukaryotic wheat germ system. In addition, we determined the expression rate, yield and solubility of those proteins. After sequence optimisation for the *E. coli in vitro *system and generating linear templates for wheat germ expression, the success rate of cell-free protein expression reached 93%.

**Conclusion:**

We have demonstrated that protein expression in cell-free systems is an appropriate technology for the successful expression of soluble full-length proteins. In our study, wheat germ expression using a two compartment system is the method of choice as it shows high solubility and high protein yield.

## Background

With the sequencing of the human genome completed and with mRNA/cDNA identification rapidly progressing, many potential novel genes have been discovered and attention has turned to the function and structure of the predicted proteins [1-4]. In order to study these novel gene products, sufficient amounts of protein generally obtained through recombinant protein expression are required. The (high-throughput) expression and characterisation of unknown and poorly characterised human proteins is a main objective of recombinant proteomic studies today.

*Escherichia coli *is the most commonly used prokaryotic expression system for the high-level production of recombinant proteins *in vivo *[5] and has already been used successfully in high-throughput protein expression and purification studies [4,6]. The use of *E. coli *has many advantages, including the ease of growth and manipulation of the organism and the availability of many different vectors and host strains that have been developed over the years. However, the use of *E. coli *also has limitations, such as the aggregation of protein in insoluble inclusion bodies, problems with the expression of gene products toxic to the physiology of the host cell or proteolytic degradation of proteins in the cytoplasm [7]. In light of these difficulties, cell-free expression systems are becoming increasingly popular [8-14]. The *in vitro *systems have several advantages, including rapid protein synthesis [15], the possibility to express toxic gene products [16] and constructs that otherwise would be proteolytically degraded. Furthermore, it is possible to express proteins with up to 10 putative transmembrane domains as reported recently [17]. The compatibility with PCR-generated templates as well as plasmids allows the *in vitro *expression reaction with *E. coli *extract to be optimised using silent mutations within PCR products [18]. These sequence optimisations reduce unfavourable secondary structures in mRNA and thus improve the success rate of translation and protein expression. In contrast, for cell-free protein expression with wheat germ lysate sequence optimisation is not necessary because of the eukaryotic nature of this source.

For protein expression analyses, a comprehensive cDNA collection is available at the German Ressource Center (RZPD). The full-length open reading frames (ORFs) are cloned into an entry vector by utilizing the Gateway^® ^cloning technology (Invitrogen). Untranslated regions are excluded and only the open reading frame is cloned into the selected vector, either with or without a stop codon. For protein expression, the open reading frame can be moved into any desired expression vector by homologous recombination. Thus, a protein can be expressed with or without a tag and the tag itself can easily be selected and altered by choosing the appropriate destination vector.

The aim of this study was to evaluate alternatives to protein expression in *E.coli in vivo *especially for those ORFs yielding no protein in this system. Therefore we investigated protein expression in two different *in vitro *systems: *E. coli *and wheat germ extract. The performance of these systems was analysed and optimised in respect to expression rate, protein yield and solubility. Altogether, we tested the expression of 960 human full-length proteins *in vivo *and *in vitro *using standardised conditions.

## Results

### Comparison of *in vivo *and *in vitro *Escherichia coli expressions

We used 960 randomly selected fully sequence-verified human open reading frames with a broad range of expected molecular weights (from less than 8 kDa up to 134 kDa, average of 35 kDa), different predicted subcellular localisations and biochemical functions including membrane proteins. The ORFs were cloned into an expression vector (pDEST17-D18), for production of proteins with an N-terminal 6xHis-tag. Identical constructs were used for protein expression *in vitro *and also for transformation of bacteria and expression *in vivo*. Protein expression was analysed by western blotting using an anti-His antibody.

In *E. coli in vivo *629 out of 960 proteins, and *in vitro *456 out of 960 proteins were successfully expressed. Protein expression in bacteria was unsuccessful either because clones were generated, which did not show protein expression (233 samples) or the transformation failed completely (98 samples). Considering the overlap of both expression systems, 206 full open reading frames yielded no protein product *in vivo *and*in vitro*. In contrast, 331 targets were expressed *in vivo *and*in vitro *with an average molecular weight of 33 kDa. Among these, 57 clones showed an expression rate of 4, 86 clones of 3 and 7 clones of 2. Furthermore, 424 targets expressed in either system with an average moleclar weight of 32 kDa.

### Optimisation of *E. coli *expression *in vitro*

**Figure 1:**
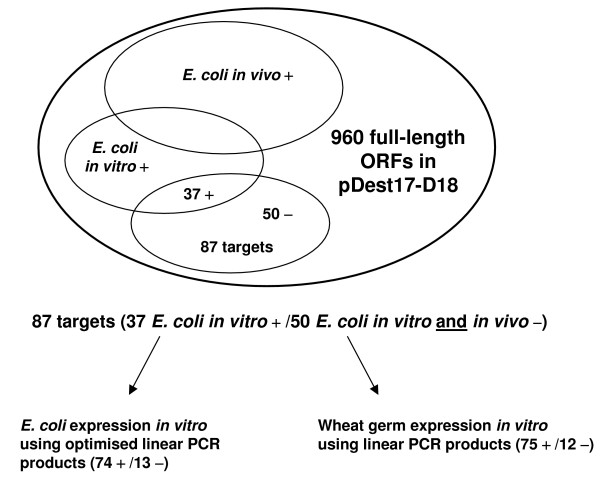
Scheme of the experimental strategy. Successful protein expression is indicated by +, unsuccessful protein expression by -.

**Figure 2:**
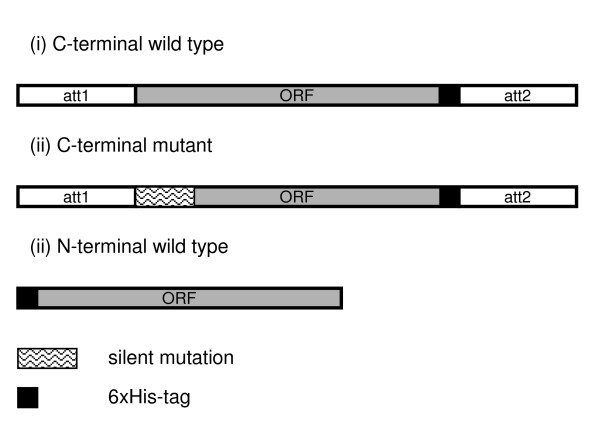
Optimised linear templates for *E. coli in vitro *expression. Three kinds of linear PCR-products were generated to investigate the effect on protein expression rate and yield. (i) C-terminal wild type with C-terminal 6xHis-tag (ii) C-terminal mutant with C-terminal 6xHis-tag and inserted silent mutation at the N-terminus (iii) N-terminal wild type with N-terminal 6xHis-tag and no attachment sites (att-sites); For the C- and N-terminal wild type template the ORF is identical to the original ORF in the plasmid DNA.

We next examined the effect of sequence optimisation on protein expression rate and protein yield by selecting randomly 87 out of the 960 ORFs (Figure 1, Table 1) where protein expression had been unsuccessful *in vivo *or where transformation had failed in BL21(DE3)pLysS. Three kinds of linear PCR templates were generated (Figure 2): (i) a C-terminal wild type with C-terminal 6xHis-tag (ii) a C-terminal mutant with C-terminal 6xHis-tag and inserted silent mutation at the N-terminus (iii) a N-terminal wild type with N-terminal 6xHis-tag and no attachment sites (att-sites); For the C- and N-terminal wild type template the ORF is identical to the original ORF in the plasmid DNA.

**Table 1:** Subset of 87 clones tested in *E. coli in vitro *and in wheat germ expression. ORF Nr.: Clone identifier; RZPD Clone ID: Available clones at RZPD GmbH; Hit Acc. Nr.: Best BLAST hit of DNA sequence. Molecular weight was calculated by translation of the DNA sequence. Expression/solubility were assigned values from 0 (no expression/no protein detectable in the supernatant) to 4 (very strong band/protein band in the supernatant is stronger than in the pellet). The column 'yielded by' is indicated as follows: WG C: wheat germ C-terminal, WG: wheat germ, C- and N-terminal, WG N: wheat germ, N-terminal, RTS CW: *E. coli in vitro *C-terminal wildtype, RTS CM: *E. coli in vitro *C-terminal mutant, RTS NW: *E. coli in vitro *N-terminal wildtype, RTS Pl: *E. coli in vitro *original plasmid.

**ORF Nr**	**RZPDCloneID**	**Hit Acc No**	**Gene symbol**	**Mw in kDa**	**Best expr. rate**	**Yielded by**	**Best solubility**
264	RZPDo834B052	NM_001677	ATP1B1	36	4	RTS NW	3
400	RZPDo834D022	NM_002573	PAFAH1B3	27	4	WG, RTS CM NW	4
433		NM_000318	PXMP3	32	4	WG, RTS CW CM NW	4
464	RZPDo834F012	NM_006793	PRDX3	29	4	WG C, RTS CM	4
505		NM_003187	TAF9	30	4	WG	3
531		NM_012222	MUTYH	23	4	WG	3
562	RZPDo834B043	NM_000075	CDK4	35	4	RTS CM NW Pl	3
571	RZPDo834B083	NM_003182	TAC1	13	4	WG C, RTS CW CM	4
616	RZPDo834D033	NM_000550	TYRP1	61	3	RTS Pl	2
636		NM_000612	IGF2	22	4	WG C, RTS CW CM	4
637	RZPDo834E013	NM_015646	RAP1B	22	4	WG, RTS CW CM NW	4
639		NM_002512	NME2	14	4	WG N, RTS NW	3
690	RZPDo834F123	NM_006923	SDF2	23	4	WG C, RTS CW CM	4
694	RZPDo834G023	NM_020470	YIF1	32	4	WG N	3
728	RZPDo834E0511	NM_000434	NEU1	46	4	RTS NW	2
741	RZPDo834H073	NM_002799	PSMB7	30	4	WG C	4
772	RZPDo834C0311	NM_002804	PSMC3	46	4	WG N, RTS CM	4
777	RZPDo834C0411	NM_003908	EIF2S2	39	4	WG	4
831		NM_004394	DAP	12	3	WG	4
832		NM_002966	S100A10	12	4	WG, RTS CM NW	4
833	RZPDo834A124	NM_017503	SURF2	30	4	WG C	4
840	RZPDo834B024	NM_005499	UBA2	72	3	WG C	4
842		NM_005942	MOCS1	24	4	WG N, RTS CM	3
855		NM_002134	HMOX2	36	4	RTS NW	4
861	RZPDo834C034	NM_006370	VTI1B	27	4	WG N, RTS NW Pl	3
868	RZPDo834C084	NM_005892	FMNL1	53	4	WG, RTS CM NW	3
873	RZPDo834C104	NM_007363	NONO	55	4	WG C	3
881		NM_002622	PFDN1	15	4	WG C	4
898	RZPDo834D114	NM_006117	PECI	21	4	WG N, RTS CW	4
901	RZPDo834E024	NM_031263	HNRPK	51	4	RTS CW NW	4
904		NM_004401	DFFA	13	4	WG N	4
906		NM_006693	CPSF4	31	4	WG C, RTS CM	4
915	RZPDo834F024	M55654	TBP	38	4	WG N, RTS NW Pl	3
918		NM_004184	WARS	54	3	WG N	4
921	RZPDo834F064	NM_004309	ARHGDIA	23	4	RTS CM NW	3
924	RZPDo834F094	NM_002861	PCYT2	44	4	WG C	4
930	RZPDo834G014	NM_001551	IGBP1	40	4	WG, RTS Pl	4
932	RZPDo834G034	NM_002070	GNAI2	41	4	WG C, RTS NW Pl	4
935		NM_001014835	PAK4	64	2	WG C	3
936	RZPDo834G064	NM_002074	GNB1	38	3	RTS NW	3
939	RZPDo834G074	NM_006321	ARIH2	58	3	WG C, RTS Pl	4
940	RZPDo834G084	NM_013296	GPSM2	55	2	WG C	4
943	RZPDo834G114	NM_001863	COX6B1	11	4	WG, RTS CW CM Pl	4
944		NM_004537	NAP1L1	46	4	WG	4
945	RZPDo834H014	NM_152925	RBM12	59	4	WG C	4
947	RZPDo834H034	NM_001017957	OS-9	70	4	WG C	4
1033		NM_007317	KIF22	74	0		0
1068		NM_206900	RTN2	52	4	WG C	4
1082		NM_018074	FLJ10374	37	4	WG; RTS CW CM	4
1091		NM_001512	GSTA4	26	4	WG	4
1093	RZPDo834A015	NM_001647	APOD	22	4	WG C, RTS CM	4
1101		NM_001643	APOA2	12	4	WG N	3
1115		NM_007261	CMRF-35H	25	4	WG, RTS CM NW	4
1189	RZPDo834H106	NM_001425	EMP3	19	4	WG	4
1294	RZPDo834A035	NM_014876	KIAA0063	24	4	WG	4
1330		NM_002816	PSMD12	53	4	WG C	4
1453	RZPDo834B115	NM_198216	SNRPB	25	4	WG C, RTS CM	3
1454	RZPDo834H0711	NM_006841	SLC38A3	56	4	WG, RTS NW	3
1461	RZPDo834C025	NM_004047	ATP6V0B	22	3	WG C	1
1462	RZPDo834C035	NM_003145	SSR2	21	4	WG C, RTS CM	3
1480		NM_000984	RPL23A	18	3	WG	4
1485	RZPDo834D025	NM_014860	SUPT7L	47	4	WG	4
1487	RZPDo834D035	NM_198120	EBAG9	25	4	WG C	3
1533	RZPDo834F075	NM_013300	HSU79274	31	4	WG C	3
1554	RZPDo834G095	NM_001778	CD48	28	4	RTS NW	3
1555	RZPDo834G105	NM_019111	HLA-DRA	29	4	RTS CM	3
1575	RZPDo834H085	NM_004233	CD83	23	4	WG, RTS CM	4
1576	RZPDo834H095	NM_007024	PL6	39	2	WG N	1
1642		NM_003490	SYN3	64	2	WG	4
1670		NM_006841	SLC38A3	56	4	WG N, RTS NW	3
1734	RZPDo834F0511	NM_001436	FBL	34	4	WG C	4
1736	RZPDo834G0511	NM_004343	CALR	49	4	WG C	4
2066		NM_015723	PNPLA8	89	0		0
2225	RZPDo834H0221	NM_018127	ELAC2	93	0		0
2229	RZPDo834A046	NM_00513	REC8L1	63	3	WG C, RTS CM	4
2504		NM_199053	FLJ12716	65	4	RTS CW CM NW	3
2724		NM_173157	NR4A1	65	4	WG N	2
2871		NM_018099	MLSTD1	60	4	RTS CW CM NW	3
2938	RZPDo834H0421	NM_015072	TTLL5	92	0		0
2949	RZPDo834E1121	NM_020748	KIAA1287	135	0		0
2959		NM_021932	RIC8	17	4	WG, RTS Pl	4
2962	RZPDo834H0621	NM_014149	HSPC049	78	2	RTS CM NW	0
2964	RZPDo834F1121	NM_003263	TLR1	91	4	RTS CM NW	4
2968	RZPDo834G0821	NM_001040428	SPATA7	65	4	RTS CW CM NW	3
2973		NM_032292	FLJ20203	91	4	RTS CW CM NW	3
2978	RZPDo834H0721	NM_014585	SLC40A1	63	0		0
2979	RZPDo834E0821	NM_013277	RACGAP1	71	4	RTS CM	0

### Influence of sequence optimisation on protein expression rate

**Figure 3:**
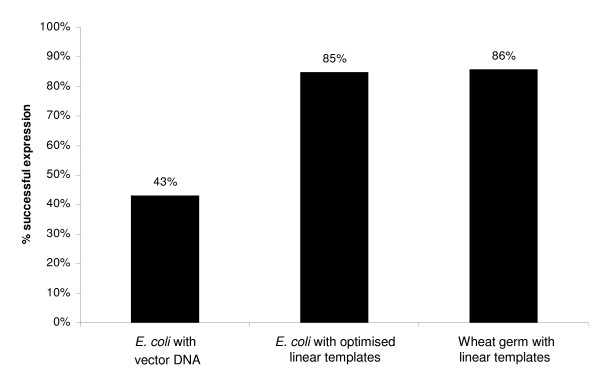
Comparison of *in vitro *expression of 87 targets in *E. coli *and wheat germ.

**Figure 4:**
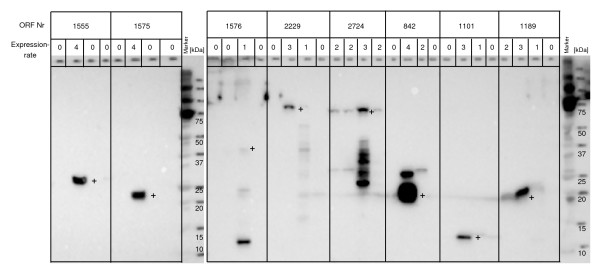
*E.coli *expression *in vitro*. Presented are western blots of 8 targets expressed with C-terminal wild type, C-terminal mutant, N-terminal wild type and original plasmid DNA templates (from left to right). Successful protein expression was defined for values 2 – 4 and unsuccessful protein expression for values of 0 and 1. Bands of the expected size are marked with a +.

Of these 87 samples, 37 samples (43%) were successfully expressed *in vitro *using the original plasmid DNA. After sequence optimisation, we increased the success rate of protein expression up to 74 samples (85%) in the cell-free *E. coli *system (Figure 3, 4).

When analysing the results of those samples which had previously not shown expression *in vitro *(50 samples), we found that following sequence optimisation 37 (74%) proteins were expressed.

### Influence of sequence optimisation on protein yield

To assess the protein expression yield of PCR products after sequence optimisation, we evaluated 37 samples that had previously expressed protein *in vitro *from original plasmid DNA. Protein yield was determined by analysis of protein bands on western blots. Bands were given marks from 0 (no expression) to 4 (very strong). Here, we discovered that 65% of expressions (24 samples) showed an improvement in the protein yield compared to expressions using original plasmid DNA and another 19% showed similar protein yields. A smaller amount of protein was expressed in only 6 cases (16%) using the optimised PCR products. Among these were 3 samples which did not express protein at all. In summary, after analysis of 87 expressions *in vitro *with optimised PCR-products, 16 samples (18%) revealed no protein product *in vivo *or *in vitro *in the *E. coli *systems (Table 2).

**Table 2:** Proteins not expressing in *E. coli in vitro *or in wheat germ or in both systems. Molecular weight was calculated by translation of the DNA sequence. Localization information was taken from the Uniprot database. Domain information was retrieved from the Pfam database: cc: coiled coil, tms: transmembrane segment, sp signal peptide. Empty fields correspond to no assignment in the database.

Hit Acc. No.	Gene symbol	Mw in kDa	Localization	Domains	Expression in *E. coli in vitro*	Expression in wheat germ
NM_007317	KIF22	74	nuclear	1cc,,	no	no
NM_015723	PNPLA8	89	membrane		no	no
NM_020748	KIAA1287	135	n/a		no	no
NM_018127	ELAC2	93	nuclear		no	no
NM_015072	TTLL5	92	n/a	3cc,,	no	no
NM_014585	SLC40A1	63	membrane	,,10tms	no	no
NM_020470	YIF1A	32	membrane	,,5tms	no	yes
NM_004394	DAP	12	secreted		no	yes
NM_001014835	PAK4	64	n/a		no	yes
NM_013296	GPSM2	55	n/a		no	yes
NM_006812	OS-9	70	n/a	1cc1sp1tms	no	yes
NM_014860	SUPT7L	47	n/a	1cc,,	no	yes
NM_003908	EIF2S2	39	nuclear		no	yes
NM_006321	ARIH2	58	nucleus	2cc,,	no	yes
NM_002816	PSMD12	53	cytosol	1cc,,	no	yes
NM_000984	RPL23A	18	cytosol		no	yes
NM_002134	HMOX2	36	microsomal	1cc,1tms	yes	no
NM_018099	MLSTD1	60	intracellular	,,2tms	yes	no
NM_013277	RACGAP1	71	intracellular	1cc,,	yes	no
NM_003263	TLR1	91	membrane	1sp,1tms	yes	no
NM_032292	FLJ20203	91	n/a	1cc,,	yes	no
NM_014149	HSPC049	78	n/a	1cc,,	yes	no

### Wheat germ expression *in vitro*

**Figure 5:**
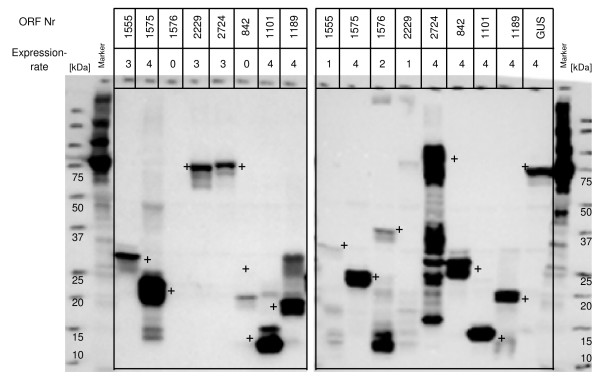
Wheat germ expression *in vitro*. Presented are western blots of 8 targets expressed with C-terminal (left) and N-terminal (right) 6xHis-tag. ORF Nr.: clone identifier. GUS: glucuronidase is the positive control. Successful protein expression was defined for values 2 – 4 and unsuccessful protein expression for values of 0 and 1. Bands of theexpected size are marked with a +.

The aim of this experiment was to elucidate whether the wheat germ system would show an increase in the success rate and protein yield of the 87 selected open reading frames compared to the optimised *in vitro *expressions in *E. coli*. Two wild type PCR constructs were made for each open reading frame, one for production of a protein with a C-terminal 6xHis-tag and another for a protein with a N-terminal 6xHis-tag (Figure 5). A total of 75 proteins could be expressed in wheat germ lysate with either a C- or a N-terminal 6xHis-tag (86%, Figure 3). Out of the 16 open reading frames which were not expressed in the *E. coli *systems, 10 were now successfully expressed using wheat germ lysate (Table 2). However, 6 open reading frames did not express in the wheat germ system, but were previously successfully expressed *in vitro *in *E. coli *(Table 2). On average, based on western blotting analyses, the protein yield was higher in the wheat germ compared to expressions in the *E. coli in vitro *system, for identical human ORFs.

### Influence of tag position on protein expression *in vitro*

**Figure 6:**
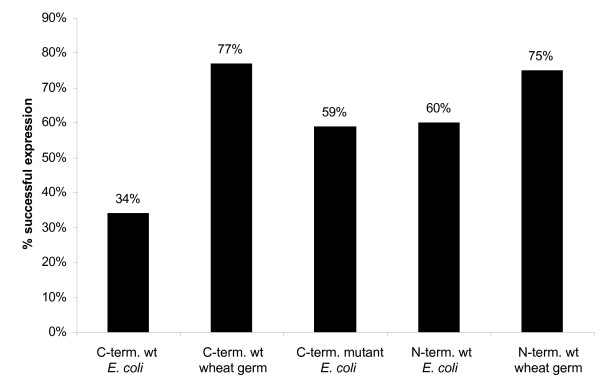
Influence of tag on *in vitro *expression. We compared 87 targets expressed in *E.coli in vitro *and in wheat germ. C-term. wt: C-terminal wild type; C-term. mutant: C-terminal mutant; N-term. wt: N-terminal wild type.

To assess the influence of either N- or C-terminal tag positions on expression rate, the 87 open reading frames were evaluated in both *in vitro *expression systems, *E. coli *and wheat germ (Figure 6). In the *E. coli in vitro *system, protein expressions using optimised PCR products were evaluated. Here, 52 (60%) N-terminal tagged wild type PCR products expressed protein compared to only 30 (34%) with C-terminal wild type PCR products (Figure 4). With the C-terminal mutant product 51 (59%) proteins were expressed. In the wheat germ system, 65 were expressed using the N-terminal wild type construct (75%) and 67 with the C-terminal tag (78%) (Figure 5).

In summary, 81 out of 87 open reading frames were expressed in both *in vitro *systems, corresponding to a success rate of 93%. Only 6 ORFs yielded no protein in any of the systems tested (Table 2).

### Comparison of solubility of proteins expressed in *E. coli in vivo*, *in vitro *and in wheat germ system

For solubility studies, the lysis supernatant of those targets revealing expression was analysed by western blot. 483 proteins, expressed in *E. coli in vivo*, were tested and 193 proteins were soluble (40%). For *E. coli *expressions *in vitro *(with original plasmid DNA), 388 were analysed and 185 proteins were soluble (48%). In the wheat germ system with a C-terminal 6xHis-tag 66 of the 68 (97%) expressing PCR products showed soluble protein and 95% with an N-terminal 6xHis-tag.

## Discussion and conclusion

With this approach we evaluated the performance of three different protein expression systems *in vivo *and *in vitro *with a set of 960 full-length open reading frames. For our investigations of protein synthesis we chose *Escherichia coli *bacteria as it is one of the most common and easy to use systems. For cell-free *in vitro *expression, we compared the *E. coli *with the wheat germ protocol.

First, we analysed the protein expression rate in the two *E. coli *systems (*in vitro *and *in vivo*) and found that expression is higher in the *in vivo *system (66% compared to 48%). Regarding the success rate of both protocols, only 22% of plasmids yielded no protein.

We then focused on a subset of 87 targets which had yielded no protein in *E. coli in vivo*. These targets expressed with the cell-free wheat germ and *E. coli *protocol yielded very different protein expression rates. In wheat germ 86% of the targets were expressed and in cell-free *E. coli *only 43%. One of the reasons for unsuccessful *in vitro *protein expression in *E. coli *may be the presence of secondary structures in mRNA, which may inhibit translation [19]. To solve this problem, we made use of the ProteoExpert software, which predicts possible sequence-related problems and proposes optimised sequences with potentially reduced unfavourable secondary structures [18,20]. Out of the 87 proteins that were not expressed in *E. coli in vivo*, 37 were expressed *in vitro *using the wild type sequence. Another set of 37 human proteins could be rescued by sequence optimisation and using linear templates for *in vitro *expression. Therefore, the overall success rate of *in vivo *negative clones was 85%. This result clearly demonstrates that sequence optimisation is necessary to improve protein synthesis in the *E. coli in vitro *system.

Furthermore, we analysed the influence of tag position on protein expression rate. We found no difference between C- or N-terminal tag in the wheat germ system. However, considering the cell-free *E. coli *system, 60% successful expression was obtained with the N-terminal wild type PCR product in contrast to only 34% with the C-terminal one. In this context it is important to realize that this 60% expression with the N-terminal tag matches with the 59% obtained with the C-terminal mutant. Obviously, modifying the sequence by adding a sequence optimised peptide tag also avoids expression problems associated with the inition of translation.

After analysis of the 87 optimised expressions *in vitro*, 6 samples remained that were not expressed *in vivo *or *in vitro *(Table 2). This corresponds to a protein expression success rate of 93%. Regarding those proteins, which could not be expressed in either system, it is striking that the molecular mass of all of these targets is higher than 63 kDa with an average molecular weight of 91 kDa. Two membrane associated proteins belong to the unsuccessful targets: PNPLA8 (89 kDa) and SLC40A1 (63 kDa), the latter with more than 10 transmembrane domains. Furthermore, a DNA binding protein KIF22 (74 kDa) of the Kinesin family, involved in spindle formation, ELAC2 (93 kDa) an endonuclease, TTLL5 (92 kDa), a tubulin tyrosin ligase-like protein and KIAA1287 (135 kDa), a hypothetical protein with one transmembrane domain, are among the non-expressing targets. At this point it is unclear whether these human proteins are functionally expressed in any of the systems. Therefore, we can not speculate about the interference between the protein function and the different expression systems.

However, 6 proteins with an average molecular weight of 72 kDa were expressed in *E. coli in vitro *but not in wheat germ. Among these proteins is HMOX2 which belongs to the heme oxygenase family, an iron-containing protein with one transmembrane domain. As reported recently, iron-containing proteins require supplemented iron sources which were not added in this case [21]. Further proteins are two with transmembrane domains (MLSTD1 with two transmembrane domains and TLR1 with one). The three proteins RACGAP1, FLJ20203 and HSPC049 each contain one coiled coil domain and have molecular weights higher than 70 kDa. Obviously, the expression of proteins with molecular weights higher than 70 kDa are critical for the wheat germ system [22].

Ten proteins with an average molecular weight of 45 kDa also remain which were expressed in wheat germ but not in *E. coli in vitro*. An explanation for this can not be found in the structural domains, because a coiled coil and one transmembrane domain were not a hindrance for expression of the proteins mentioned before. Also the molecular weight is not the problem. Regarding the function of these proteins, SUPT7L (transcription regulation factor), EIF2S2 (translation initiation factor) and RPL23A (rRNA binding protein) are proteins which interfere with DNA or RNA. It seems that those proteins are likely to have negative effects on their recombinant expression, when functional active in *E.coli *cells. Also proteins influencing the cell cycle like DAP (involved in cell death), KIAA1142 (has a kinase motif), PAK4 (kinase, involved in the JNK pathway), GPSM2 (a signalling modulator) and OS-9 (influences cell growth viability) seem to hamper recombinant protein expression.

Based on western blotting analyses, the protein yield in wheat germ was higher compared to expressions in the *E. coli in vitro*. This may be due to the fact that the *in vitro E. coli *expression system is a batch method for protein expression, whereas the wheat germ system is based on a two-compartment system. The two chambers are separated by a semi-permeable membrane which concentrates the expressed protein in the 50 μl reaction chamber, but lets compounds required for protein synthesis such as substrates and energy components pass through into the larger feeding chamber. At the same time, potentially inhibitory by-products are diluted via diffusion across the membrane. The wheat germ system showed the highest rate of success compared to expression in *E. coli in vitro *or *in vivo*. Thus, for *in vitro *protein expression, specifically for toxic proteins which can not be expressed in bacteria, the wheat germ system is the method of choice.

Comparing protein solubility in *E. coli *bacteria and the cell-free *E. coli *and wheat germ systems, we found that the wheat germ system produces the highest solubility rate (97%). This was also reported previously [22]. It should be mentioned that our experimental procedure does not exclude the formation of protein aggregates. Moreover, the data show that the proteins expressed *in vitro *are more likely to be soluble than those expressed *in vivo*. However, even though the *E. coli in vivo *expressions showed, in a first approach, a higher success rate than *in vitro*, the *in vitro *system does have advantages. Protein expression is very fast and can be accomplished within a few hours. The expression of toxic gene products allows proteins to be expressed, which are impossible to express in bacteria. Also the use of PCR products is possible, and no clones are necessary for protein expression. However, linear DNA needs to be protected during the in vitro reactions to suppress nuclease activity. In addition, proteins are also more likely to be soluble when expressed in any of the *in vitro *systems used compared to expression in bacteria.

In summary, we have demonstrated that cell-free protein expression leads to the desired full-length protein with an overall success rate of up to 93%. In our study, wheat germ expression using a two compartment system is the method of choice as it shows high solubility and high protein yield.

## Methods

### Expression-vector construction

The genes used in this study are available from the RZPD full-ORF clone collection. Entry clones containing the genes of interest were generated by utilising the Gateway^® ^Cloning technology (Invitrogen). All entry clones were fully sequenced in order to verify the insert within pDONR201. From the entry clone, the ORF was sub-cloned to a Gateway^® ^destination vector (pDEST17-D18, a modification of pDEST17, Invitrogen) creating an expression clone (LR reaction), which was then transformed into DH10B bacteria. Plasmid DNA of individual clones was used for transformation of BL21 (DE3) pLysS bacteria and for protein expression *in vivo *as well as for protein expression in the cell-free *E. coli *system. The pDEST17-D18  destination vector was used to express selected recombinant proteins controlled by the T7 promoter with an N-terminal 6xHis-tag. Identical constructs were used for protein expression in *E. coli *as well as for expressions in the cell-free *E. coli *system. All DNA preparations were carried out by a Qiagen Biorobot 9600 using Qiawell 96 Ultra Plasmid Kits (Qiagen).

### *In vivo *protein expression using *E. coli *bacteria

Competent BL21 (DE3) pLysS (Novagen) bacteria were transformed with plasmid DNA (pDEST17-D18 containing the gene of interest). The generated expression clones were cultured overnight, diluted 1:50 to a final volume of 3 ml, and incubated in 24-well plates at 30°C or 3.5 h (until the OD_600 _was 0,4–0,6). Expression was induced with 1 mM IPTG and bacteria cultured for a further 3,5 h at 30°C. Cells were harvested by centrifugation. A 5 μl aliquot of cell-pellet was removed and added to 45 μl of SDS sample buffer. 10 μl of the sample were then loaded onto a gel for western blotting analysis. An aliquot of the original sample was also saved for analysis of protein solubility.

### *In vitro *protein expression (*E. coli*) using vector DNA

*In vitro *protein expression was carried out using pDEST17-D18 plasmid DNA containing the ORF of interest. A cell-free batch expression system (RTS 100 *E. coli *HY kit, Roche Diagnostics) was utilised and 50 μl reactions were prepared according to the manufacturer's instructions. In brief, the samples were incubated at 30°C for 4 hours in a thermal cycler. Green fluorescent protein was expressed as control protein. Following incubation, a 5 μl aliquot was removed and added to 45 μl of SDS sample buffer. 10 μl of sample were then loaded onto a gel for Western blotting analysis. An aliquot of the original sample was also saved for analysis of protein solubility.

### *In vitro *protein expression (*E. coli*) using optimised linear PCR products

Three PCR products were created for each ORF, a C-terminal wild type, a C-terminal mutant and a N-terminal wild type product (Figure 2). Sequence-verified templates were applied for the amplification of PCR products with the Linear Template Generation Set (LTGS, Roche Diagnostics). For the C-terminal mutant template, silent mutations as proposed by ProteoExpert  were introduced at the N-terminus of the sequence. PCR was performed using partially matching primers along the first 15 to 20 nucleotides of each ORF. One gene-specific sense primer containing silent mutations, one gene-specific anti-sense and one wild type primer were used to produce the first PCR product. Different primers were applied depending on whether a C- or a N-terminal 6xHis-tag was desired. The PCR products were checked on agarose gels, and the second amplification step was carried out according to the supplier's instructions. As positive control protein, green fluorescent protein was expressed. Prior to *in vitro *expression, all products were verified for correct size and purity. *In vitro *expression was carried out according to instructions and SDS samples prepared.

### *In vitro *protein expression (wheat germ) using linear PCR products

Specific PCR products were generated to achieve translation in wheat germ lysate. The first wild type PCR product generated for optimisation in the *E. coli in vitro *system was utilised to produce a second PCR product for the wheat germ system. Linear templates with a T7 promoter and a Kozak sequence were generated for protein expression in wheat germ lysate. In contrast to PCR products created for the *E. coli in vitro *system, these products did not contain silent mutations. The first PCR products were made using gene-specific primer pairs and the second amplification step was carried out by the RTS Wheat Germ LTGS kit (Roche Diagnostics) according to instructions. The PCR products were again checked for correct size and purity. Proteins were expressed using the RTS 100 Wheat Germ CECF kit (Roche Diagnostics, positive control: glucuronidase) and contained either a C- or an N-terminal 6xHis-tag. Samples (50 μl) were incubated at 24°C, 900 rpm for 24 h (ProteoMaster Instrument, Roche Diagnostics), SDS samples prepared for western blotting and an aliquot saved for analysis of protein solubility.

### Analysis of protein solubility

An aliquot of the induced bacterial culture was mixed with a lysis reagent (Pop Culture Reagent, Novagen) and 0.1% Tween 20 and incubated for 10 min at room temperature. The sample was centrifuged at 10000 g for 20 min, the supernatant and the pellet were separated and SDS samples prepared for western blotting analysis. For the *in vitro *systems, samples were centrifuged directly and the pellet and supernatant separated. Results were expressed as values ranging from 0 (no protein detectable in the supernatant) to 4 (the protein band in the supernatant is stronger than in the pellet). Values of 0 to 1 were defined as insoluble and values of 2 to 4 as soluble protein. Values correspond to: 4 > 70%; 3 > 40%; 2 > 10%; 1 < 10% solubility; 0 = unsoluble.

### Western blotting

Western blotting was performed with the Criterion System (BioRad) and 10–20% gradient pre-cast gels. Samples (10 μl) were heated at 95°C for 5 min and loaded onto the gel, which was run at 200 V, 400 mA for 1 h. Following electrophoresis, gels were blotted onto PVDF membranes (Hybond P, Amersham Pharmacia) at 100 V, 1000 mA for 1 h and protein transfer checked by briefly immersing the membrane in Ponceau S solution (Sigma). Membranes were thoroughly washed in TBST (2 mM Tris/HCl, pH 7.6; 13.7 mM NaCl and 0.1% (v/v) Tween 20) and then blocked for 1 h in 5% (w/v) non-fat milk/TBST. Following another 3 × 15 min washes in TBST, membranes were incubated with the anti-His mouse antibody (Qiagen, 1:2000 in 3% (w/v) bovine serum albumin/TBST) overnight at 4°C. Following incubation with the secondary antibody (Anti-mouse IgG HRP, Southern Biotech) for 1 h, membranes were washed three times in TBST and developed with ChemiGlow^® ^(Alpha Innotech) chemiluminescent substrate for 5 min. Images were obtained using a CCD camera system (ChemiImager 5500, Alpha Innotech). Protein bands on western blots were assigned values from 0 (no expression) to 4 (very strong band). Successful protein expression was defined for values of 2 to 4 and unsuccessful expression for values of 0 and 1. The ratings reflect the relative amount of human fusion protein compared to the reference protein (positive control). 4 ≥ reference protein; 3 ≥ 50% of r. p.; 2 ≥ 10% of r. p.; no expression <1< 10% of r.p.; 0 = no expression.

## Authors' contributions

CL coordinated the experiments and helped to draft the manuscript. BG drafted the manuscript. NW and TS performed the experiments. LE built the database. JL provided plasmids. BK organised funding and helped to draft the manuscript and coordinated the study. All authors read and approved the final manuscript.
